# Supplementary material for: Orthophosphate increases the efficiency of slow muscle-myosin isoform in the presence of omecamtiv mecarbil
Source: Nat Commun. 2020 Jul 7;11:3405. doi: 10.1038/s41467-020-17143-2 (PMC7341760; doi:10.1038/s41467-020-17143-2)
Supplement: Supplementary file 1 — Supplementary Information [file 41467_2020_17143_MOESM1_ESM.pdf]

Orthophosphate increases the efficiency of slow muscle-myosin isoform in the presence of omecamtiv mecarbil

Governali *et al.*

Supplementary Information

Supplementary Note 1

Estimate of the effect of Pi on half-sarcomere stiffness.

The effect of increase in [Pi] (range 1-30 mM) on the half-sarcomere stiffness (k_0) in control and in the presence of 1 μM OM has been defined by determining the T_1 relation[1] (Supplementary Figure 1). In control the increase in [Pi] to 10 mM decreases T_0 (points on the ordinate of Supplementary Figure 1a: circle, without added Pi; triangle, 10 mM Pi) and the abscissa intercept of the T_1 relation, the hs strain Y_0, from 156 ± 1 kPa and 5.4 ± 0.2 nm to 110 ± 2 kPa (-30%) and 4.7 ± 0.3 nm (-13%) respectively. Thus Y_0 in 10 mM Pi reduces less than in proportion to force indicating a reduction in the slope of the T_1 relation, that is the half-sarcomere stiffness k_0, from 29.2 ± 1.2 kPa nm^{-1} (no added Pi) to 24.4 ± 1.7 kPa nm^{-1} (-16%) (10 mM Pi). In the presence of 1 μM OM the inhibitory effect of the rise in Pi on force is absent for [Pi] < 10 mM and becomes evident only for [Pi] > 10 mM, while the reduction in k_0 occurs also in the range of [Pi] increase from 1 to 10 mM: T_0 is 77 ± 4 kPa in solution without added Pi and 74 ± 1 kPa in 10 mM Pi (points on the ordinate of Supplementary Figure 1b: circle, without added Pi; triangle, 10 mM Pi). The corresponding k_0 values are 30.2 ± 1.9 kPa nm^{-1} and 23.2 ± 0.4 kPa nm^{-1} (-24%). Data for [Pi] up to 30 mM are summarized in Supplementary Table 1.

The stiffness of the motor array e_0 and the average strain per motor s_0 in control and in the presence of 1 μM OM at the different [P_i] are calculated according to Equations (1) and (2) (see Methods) from k_0 reported in Supplementary Table 1 and C_f (15.2 nm MPa^{-1}).

Supplementary Note 2.

Model calculation.

The system of linear differential equations describing the kinetics of the myosin-actin interaction cycle during isometric contraction in Fig. 6 is:

dAM'(t)/dt = k_{-1}*M.ATP(t) + k_{+5}*AM'.ADP - (k_{+1}*[ATP] + k_{-5}*[ADP])*AM'(t);

dM.ATP(t)/dt = k_{+1}*[ATP]*AM'(t) + k_{-2}*M.ADP.Pi(t) + k_{+7}*M^{*}.ADP.Pi(t) + k_{+13}*M''$_{OM}$.ADP – (k_{-1} + k_{+2} + k_{-7} + k_{-13})*M.ATP(t)

dM.ADP.Pi(t)/dt = k_{+2}*M.ATP(t) + k_{-3}*AM'.ADP.Pi(t) + k_{-8}*M$_{OM}$.ADP.Pi(t) – (k_{-2} + k_{+3} + k_{+8}*[OM])*M.ADP.Pi(t)

dAM'.ADP.Pi(t)/dt = k_{+3}*M.ADP.Pi(t) + k_{-4}*[Pi]*AM'.ADP(t) + k_{-6}*M^{*}.ADP.Pi + k_{+14}*[Pi]*AM''$_{OM}$.ADP – (k_{-3} + k_{+4} + k_{+6} + k_{-14}*[OM])*AM'.ADP.Pi(t)

dAM'.ADP(t)/dt = k_{+4}*AM'.ADP.Pi(t) + k_{-5}*ADP*AM'(t) – (k_{-4}*[Pi] + k_{+5})*AM'.ADP(t)

dM^{*}.ADP.Pi(t)/dt = k_{+6}*AM'.ADP.Pi(t) + k_{-7}*M.ATP(t) - (k_{-6} + k_{+7})*M^{*}.ADP.Pi(t)

dM$_{OM}$.ADP.Pi(t)/dt = k_{-9}*AM'$_{OM}$.ADP.Pi(t) + k_{+8}*[OM]*M.ADP.Pi(t) - (k_{+9} + k_{-8})*M$_{OM}$.ADP.Pi(t)

dAM'$_{OM}$.ADP.Pi(t)/dt = k_{+9}*M$_{OM}$.ADP.Pi(t) + k_{-10}*[Pi]*AM'$_{OM}$.ADP(t) – (k_{-9} + k_{+10})*AM'$_{OM}$.ADP.Pi(t)

dAM'$_{OM}$.ADP(t)/dt = k_{+10}*AM'$_{OM}$.ADP.Pi(t) + k_{-11}*AM''$_{OM}$.ADP(t) – (k_{-10}*[Pi] + k_{+11})*AM'$_{OM}$.ADP(t)

dAM''$_{OM}$.ADP(t)/dt = k_{+11}*AM'$_{OM}$.ADP(t) + k_{-12}*M''$_{OM}$.ADP(t) + k_{-14}*[OM]*AM'.ADP.Pi(t) – (k_{-11} + k_{+12} + k_{+14}*[Pi])*AM''$_{OM}$.ADP(t)

dM''$_{OM}$.ADP(t)/dt = k_{+12}*AM''$_{OM}$.ADP(t) + k_{-13}*M.ATP(t) – (k_{-12} + k_{+13})*M''$_{OM}$.ADP(t)

where [ATP] is 5 mM and [ADP] is 20 μM. Values of the rate constants are listed in Table 3. k_{-7} = k_{-13} = 0. Force is calculated by the sum of the occupancies of the AM'ADP.Pi and AM'ADP states. Differential equations are solved by numerical integration by means of Euler method using LabView (NI) software.

Supplementary Figures

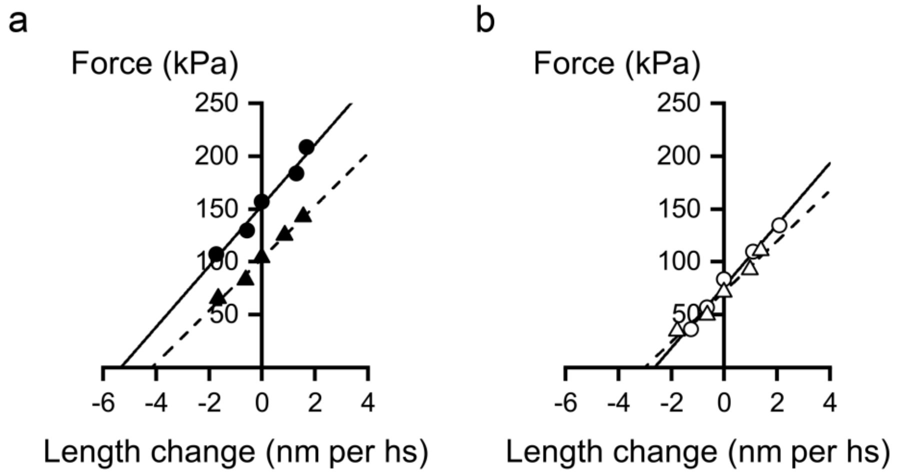

Supplementary Figure 1. Effect of Pi on the half-sarcomere stiffness estimated by the slope of T_1 relation. Data from a fibre in control (a) and in the presence of 1 μM OM (b). Circles: T_1 relations in the absence of added Pi; triangles: T_1 relations in the presence of 10 mM added Pi. Lines are linear regression equations fitted to the circles (continuous lines) and triangles (dashed lines), and their slopes measure the half-sarcomere stiffness. Source data are provided as a Source Data file.

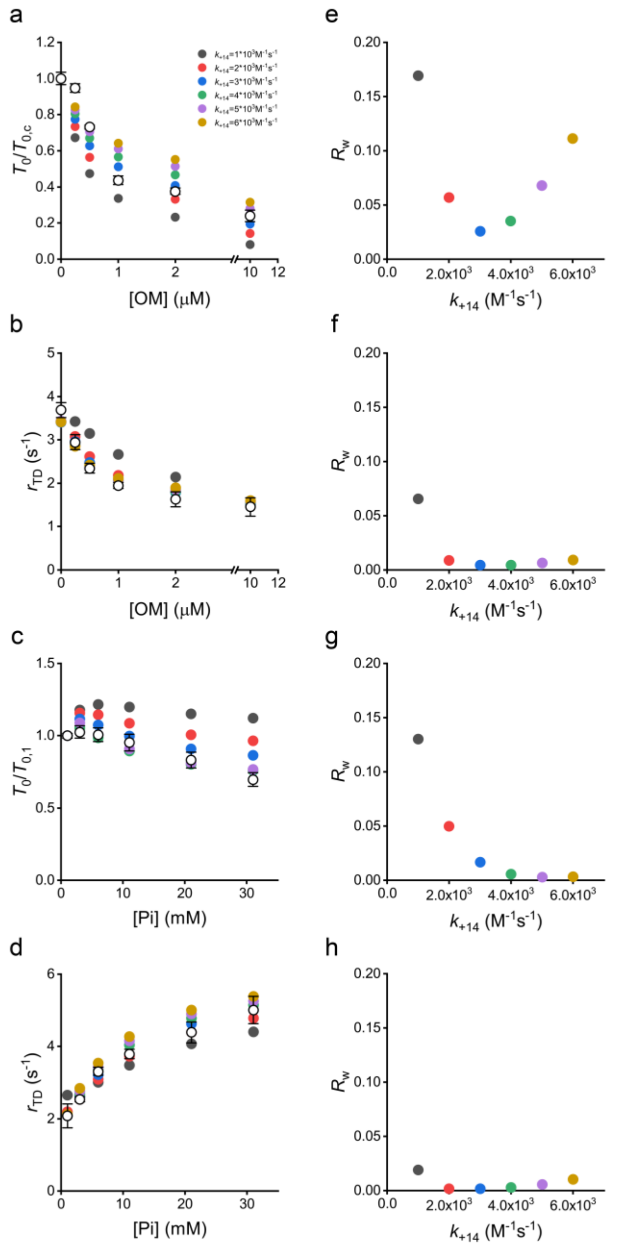

Supplementary Figure 2. Selection procedure for the forward rate constant of step 14 (k_{+14}). Left column: open circles, experimental relations of the isometric force versus [OM] (a, data from Fig. 1c, with force relative to the value without OM, mean values ± SEM from eight fibres), r_{TD} versus [OM] (b, same data as in Fig.1d), force versus [Pi] in 1 μM OM (c, same data as in Fig. 4a open circles), and r_{TD} versus [Pi] in 1 μM OM (d, same data as in Fig. 4b open circles); filled circles, simulated relations obtained by changing k_{+14} according to the colour code in the inset. As detailed in Methods, the same procedure is applied to other four relations for a total of eight relations. The four relations not shown for simplicity are: the stiffness of the motor array (e_0) versus either [OM] (data from Fig. 2f) or [Pi] (data from Fig. 4c open circles) and k_{cat} versus either [OM] (data from Fig. 5b) or [Pi] (data from Fig. 5d). Right column, plots e-h: mean values of the weighted residuals (R_w, see Methods) versus the value of k_{+14} assumed in the simulation, identified by the same colour code as in the corresponding relation in the same raw of the left column.

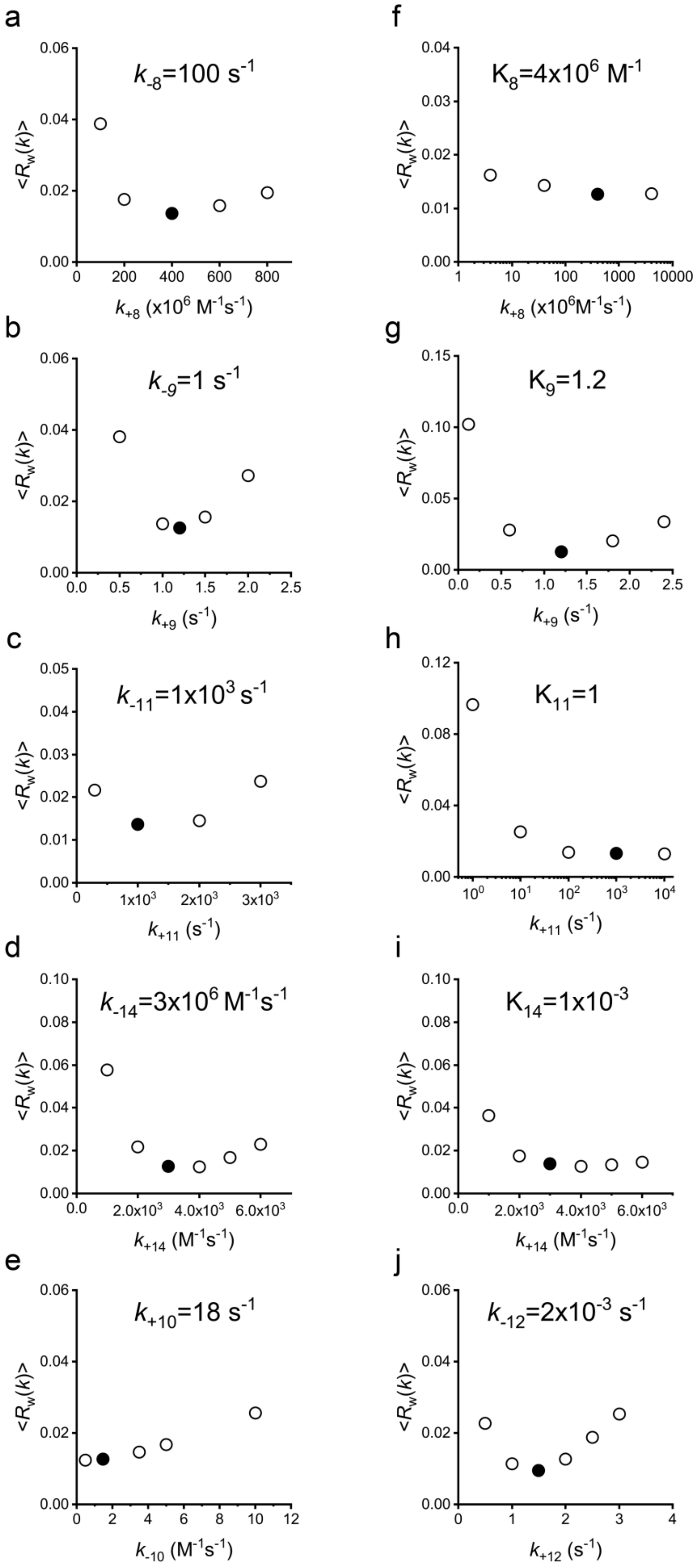
a
k_{-8}=100 s^{-1}
<$R_w(k)$>
k_{+8} (x10^6 $M^{-1}s^{-1}$)
b
k_{-9}=1 s^{-1}
k_{+9} (s^{-1})
c
k_{-11}=1x10^3 s^{-1}
k_{+11} (s^{-1})
d
k_{-14}=3x10^6 $M^{-1}s^{-1}$
k_{+14} ($M^{-1}s^{-1}$)
e
k_{+10}=18 s^{-1}
k_{-10} ($M^{-1}s^{-1}$)
f
K_8=4x10^6 M^{-1}
k_{+8} (x$10^6M^{-1}s^{-1}$)
g
K_9=1.2
k_{+9} (s^{-1})
h
K_{11}=1
k_{+11} (s^{-1})
i
K_{14}=1x10^{-3}
k_{+14} ($M^{-1}s^{-1}$)
j
k_{-12}=2x10^{-3} s^{-1}
k_{+12} (s^{-1})

Supplementary Figure 3. Global mean of the weighted residuals from all the eight relations ($< Rw\ (k) >$, see Methods) versus the rate/equilibrium constants under consideration. The left column from a to d refers to changes of only the forward rate constant, as indicated by the abscissa, and the right column from f to i refer to changes of both rate constants, so that the equilibrium constant of the step identified by the row remains the same. e and j refer to changes of k_{-10} (given the constraint for k_{+10} according to Ref (9,15), see Table 3) and k_{+12} respectively. In each panel the label reports the value of the rate/equilibrium constant maintained constant during the iteration. In each plot the abscissa of the filled circle is the value of the parameter under inspection selected for Table 3.

Supplementary Tables

Added [Pi] (mM)	T_0 (kPa)	k_0 (kPa nm^{-1})	Y_0 (nm)	e_0 (kPa nm^{-1})	s (nm)
(A)					
0	156 ± 1	29.2 ± 1.2	5.41 ± 0.20	54.2 ± 4.4	3.08 ± 0.19
2.5	135 ± 1	26.6 ± 1.2	5.12 ± 0.23	45.1 ± 3.5	3.09 ± 0.23
5	123 ± 3	25.6 ± 1.8	4.89 ± 0.31	43.2 ± 5.8	3.04 ± 0.30
10	110 ± 2	24.4 ± 1.7	4.68 ± 0.29	39.8 ± 4.5	2.95 ± 0.30
20	92 ± 1	20.6 ± 1.3	4.55 ± 0.30	30.6 ± 2.7	3.19 ± 0.29
30	83 ± 1	18.1 ± 1.0	4.67 ± 0.19	25.1 ± 1.9	3.36 ± 0.23
(B)					
0	77 ± 4	30.2 ± 1.9	2.44 ± 0.29	57.5 ± 7.4	1.41 ± 0.15
2.5	78 ± 2	28.5 ± 1.6	2.85 ± 0.18	51.2 ± 5.0	1.59 ± 0.18
5	80 ± 1	27.2 ± 1.5	2.86 ± 0.21	47.0 ± 4.2	1.77 ± 0.18
10	74 ± 1	23.2 ± 0.4	3.18 ± 0.12	35.9 ± 1.0	2.06 ± 0.06
20	63 ± 2	21.7 ± 1.1	2.88 ± 0.12	32.7 ± 2.7	1.97 ± 0.14
30	55 ± 1	20.3 ± 0.5	2.70 ± 0.04	29.5 ± 0.8	1.87 ± 0.04

Supplementary Table 1. Dependence on Pi of isometric force (T_0), hs stiffness (k_0), hs strain (Y_0), cross-bridge stiffness (e_0), and cross-bridge strain (s) in control (A) and in the presence of 1 µM OM (B). Data are mean ± SEM from three fibres. Source data are provided as a Source Data file.

A								
	Na_2ATP	$MgCl_2$	EGTA	HDTA	CaEGTA	TES	Na_2CP	GSH
Relaxing	5.4	7.7	25	-	-	100	19.1	10
Preactivating	5.5	6.9	0.1	24.9	-	100	19.5	10
Activating	5.5	6.8	-	-	25	100	19.5	10
**B**								
	Na_2ATP	$MgCl_2$	EGTA	HDTA	CaEGTA	TES	GSH	KH_2PEP
Relaxing	5.4	8.3	25	-	-	100	10	16
Preactivating	5.4	8.0	0.1	24.9	-	100	10	16
Activating	5.5	7.7	-	-	25	100	10	16

Supplementary Table 2. Composition of solutions in control (0 added Pi).
A, solutions used for sarcomere level mechanical experiments. B, solutions used for ATPase measurements. To measure the ATPase, pyruvate kinase (4 mg ml^{-1}) and lactate dehydrogenase (0.25 mg ml^{-1}) were added to the solutions. The composition of relaxing, preactivating and activating solutions with different Pi concentrations was obtained by changing CaEGTA and adjusting Na_2CP to keep the ionic strength constant. All concentrations are in mM. ATP, adenosine 5'-triphosphate; EGTA, ethylene glycol-bis-(b-aminoethyl ether)-N,N,N',N'-tetraacetic acid; HDTA, 1,6 diaminohexane-N,N,N',N'-tetraacetic acid; TES, N-tris[hydroxymethyl]methyl-2-aminoethanesulphonic acid; CP, N-[Imino(phosphonoamino) methyl]-N-methylglycine; GSH, glutathione, PEP, Phospho(enol)pyruvic acid monopotassium salt**.** 1 mg ml^{-1} creatine phosphokinase, 10 mM trans-epoxysuccinyl-L-leucylamido-(4-guanidino)butane (E-64) and 20 mg ml^{-1} leupeptin, were added to all solutions. In all solutions, ionic strength ranged between 188 and 195 mM, free Mg^{2+} was 1.2 mM and MgATP was 5 mM. In activating solution pCa ranged between 4.8 and 4.4. pH (adjusted with KOH) was 7.1 at 15 °C. All chemicals were obtained from Sigma (St. Louis, MO).

Supplementary References

1 Linari M, Caremani M, Piperio C, Brandt P, Lombardi V. Stiffness and fraction of Myosin motors responsible for active force in permeabilized muscle fibers from rabbit psoas. *Biophys J* **92**, 2476-2490 (2007).
